# Spaceflight-Associated Vascular Remodeling and Gene Expression in Mouse Calvaria

**DOI:** 10.3389/fphys.2022.893025

**Published:** 2022-05-13

**Authors:** Jamila H. Siamwala, Brandon R. Macias, Robert Healey, Brett Bennett, Alan R. Hargens

**Affiliations:** ^1^ Department of Orthopedic Surgery, University of California, San Diego, San Diego, CA, United States; ^2^ Department of Molecular Physiology, Pharmacology and Biotechnology, Brown University, Providence, RI, United States; ^3^ Vascular Research Laboratory, Providence Veterans Affairs Medical Center, Providence, RI, United States; ^4^ KBRwyle, Houston, TX, United States; ^5^ Association of Spaceflight Professionals, St. Petersburg, FL, United States

**Keywords:** osteogenesis, angiogenesis, BMP-2, VEGFA, spaceflight, long-duration flight, gene expression

## Abstract

Astronauts suffer from a loss of bone mass at a rate of 1.5% per month from lower regions of the body during the course of long-duration (>30 days) spaceflight, a phenomenon that poses important risks for returning crew. Conversely, a gain in bone mass may occur in non-load bearing regions of the body as related to microgravity-induced cephalad fluid shift. Representing non-load bearing regions with mouse calvaria and leveraging the STS-131 (15-day) and BION-M1 (30-day) flights, we examined spatial and temporal calvarial vascular remodeling and gene expression related to microgravity exposure compared between spaceflight (SF) and ground control (GC) cohorts. We examined parasagittal capillary numbers and structures in calvaria from 16 to 23 week-old C57BL/6 female mice (GC, n = 4; SF, n = 5) from STS-131 and 19–20 week-old C57BL/6 male mice (GC, n = 6; SF, n = 6) from BION-M1 using a robust isolectin-IB4 vessel marker. We found that the vessel diameter reduces significantly in mice exposed to 15 days of spaceflight relative to control. Capillarization increases by 30% (SF vs. GC, *p* = 0.054) in SF mice compared to GC mice. The vessel numbers and diameter remain unchanged in BION-M1 mice calvarial section. We next analyzed the parietal pro-angiogenic (*VEGFA*) and pro-osteogenic gene (*BMP-2, DMP1, RUNX2* and *OCN*) expression in BION-M1 mice using quantitative RT-PCR. *VEGFA* gene expression increased 15-fold while *BMP-2* gene expression increased 11-fold in flight mice compared to GC. The linkage between vascular morphology and gene expression in the SF conditions suggests that angiogenesis may be important in the regulation of pathological bone growth in non-weight bearing regions of the body. Short-duration microgravity-mediated bone restructuring has implications in planning effective countermeasures for long-duration flights and extraterrestrial human habitation.

## Introduction

Gravity-dependent retrograde blood flow to upper non-load bearing regions and blood pooling in the brain are linked to increased facial edema, cerebral venous pressure, intracranial blood volume, intracranial cerebrospinal fluid volume (CSF) and stroke volume (Morey and Baylink, 1978; Collet et al., 1997; Miyamoto et al., 1998; Iwamoto et al., 2005; Arbeille et al., 2015; Marshall-Goebel et al., 2019). In addition, altered cerebral hemodynamics account for neurological symptoms common in astronauts that include visual impairment and intracranial pressure (VIIP) syndrome, post-flight headaches, syncope, dizziness, and impaired cognitive abilities (Blaber et al., 2011; Mader et al., 2011). The skull is in close proximity to cerebral vessels impacted by microgravity and acts as a stress absorber (Tanaka et al., 2004). Although several spaceflight studies have examined the effects of prolonged shifts in hydrostatic gradients in the load-bearing, lower regions of the body (Morey and Baylink, 1978; Miyamoto et al., 1998; Vico et al., 2000; Gerbaix et al., 2018), few studies have examined vascular structural modifications and gene expression in skull bone, also known as calvaria (Kusumbe et al., 2014; Ramasamy et al., 2014). Calvarial syndesmotic sutures are active regions of growth, movement, and repair (Zimmermann et al., 1998). During development, vascularization precedes early osteogenesis on the thin plate of the skull and is followed by the formation of a plate caudal to the first. In addition, resident stem cells or osteoprogenitor cells in the suture mesenchyme contribute to the repair of stressed or fractured calvarial bones by secreting Vascular Endothelial Growth Factor (VEGF) and recruiting blood vessels and chondroclasts (Doro et al., 2017). In pathological condition or synostosis, the sutures close prematurely resulting in skeletal malformations seen in genetic disorders (Bellus et al., 1996). Clinically, in children with a small skull base, abnormal calvarial growth and cerebral perfusion increases intracranial pressure (ICP), a trend seen in astronauts (Pierre-Kahn et al., 1980; Sainte-Rose et al., 1984). 30 days of spaceflight have been shown to exacerbate these processes, where an elevation of brain arterial flow increases stiffness, myogenic vasoconstriction, and maximal diameter of the cerebral arteries as mediated by voltage-gated Ca^2+^ mechanisms (Sofronova et al., 1985; Taylor et al., 2013). Our previous work showed a 30% increase in calvarial bone mineral density in mice after 15 days of microgravity on the STS-131 flight (Zhang et al., 2013).However, there are few reports on bone vascular adaptations to microgravity at a multi-scale level (Sofronova et al., 1985; Taylor et al., 2013).

Based on the state-of-the-research, we propose that calvarial bone and vascular remodeling occurs in response to skeletal unloading, cephalad fluid shifts, and elimination of gravity-oriented hydrostatic gradients. However, the exact adaptation of calvarial vasculature to cephalad fluid shifts is not yet fully characterized. In this line of inquiry, we examine the adaptations in vascular morphology, capillarization, and osteogenic and angiogenic gene expression in mouse calvaria exposed to microgravity using image morphometry and quantitative polymerase chain reaction (qPCR). We also provide a comprehensive protocol for the isolation of RNA from thick skull bone tissue in quantities sufficient for determining calvarial angiogenesis and osteogenesis-associated gene responses.

## Materials and Methods

**FIGURE 1:**
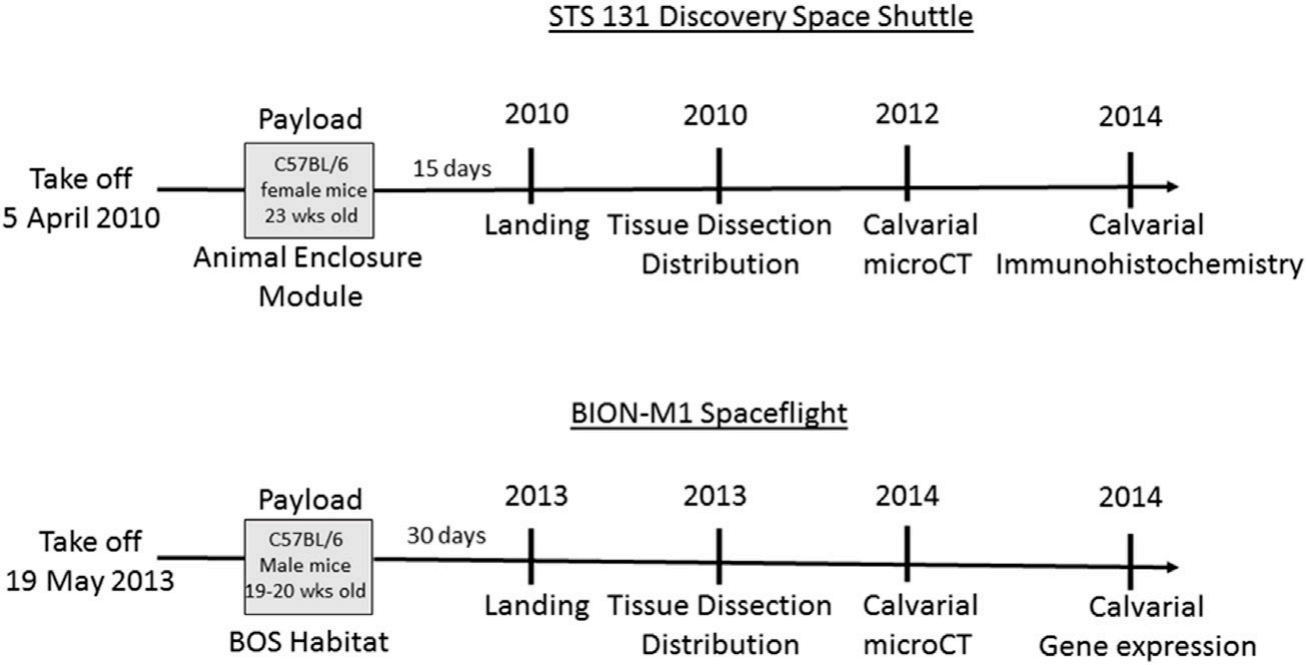
Schematic of the timeline for STS-131 and BION-M1 missions and associated design of spaceflight experiments. The Discovery vehicle launched for STS-131 on 5 April 2010 carrying 23 week-old C57BL/6 female mice. At the conclusion of a 15-day orbital flight, Discovery landed and experimental subjects were collected. Following euthanization, mouse tissue was dissected and distributed to participating laboratories. We received the mouse calvaria and performed µCT and immunohistochemistry analysis on the calvaria. Next, 19–20 week-old male mice were aboard the BION-M1 spaceflight for 30 days before landing, tissue dissection and distribution. The mouse calvaria were transported in RNA later to our lab for µCT, histochemistry, and gene expression analysis.

Experiments were performed on two groups of mice, one flown on NASA’s STS-131 flight for 15 days and the other on the BION-M1 flight for 30 days, and each spaceflight (SF) cohort was associated with a terrestrial ground control (GC). The time scale from loading the mice on Earth, landing, distribution of tissue and calvarial measurements for mice from both flights are illustrated in Figure 1 (Panels A and B). The study was approved by the Biomedical Ethics Committee of the Russian Federation State Research Center Institute for Biomedical Problems (IBMP) as well as the IACUCs of Moscow State University and NASA. The experimental protocols were conducted in compliance with the European Convention for the Protection of Vertebrate Animals used for Experimental and Other Scientific Purposes. Kennedy Space Center (KSC) veterinarians certified all STS-131 mice as healthy prior to flight, while BION-M1 mice were purchased from the Animal Breeding Facility Branch of Shemyakin and Ovchinnikov Institute of Bioorganic Chemistry.

### Animals

#### STS-131 Flight

The STS-131 flight included eight C57BL/6 female mice (16–23 week-old; Charles River, Raleigh, NC, United States). Age-matched GC mice were housed in a 12-h light-dark cycle in an Animal Enclosure Modules situated in Discovery’s middeck and provided with pre-adapted food and water ad libitum. All the mice were weighed before cage loading and again after cage unloading before euthanizing the mice on the ground. After 15 days, STS-131 returned to NASA-KSC and SF mice were distributed to multiple investigators, euthanized, and tissues harvested within 3–4 h. The Biospecimen Sharing Project (BSP), enabled mice tissue sharing among different investigators from different campuses.

### BION-M1 Flight

Of the eleven, 19–20 week-old male wild-type, pathogen-free C57BL/6 mice, five were flown on the BION-M1 biosatellite. The remaining five mice comprising the GC were maintained terrestrially. Both SF and GC groups were housed under identical conditions in Russian Block Obespecheniya Soderzhaniya (BOS) habitats on a 12-h light-dark cycle in identical environmental conditions and consumed a paste food diet containing 74.6% H_2_O developed by Russia’s IBMP. SF mice were loaded onto the unmanned BION-M1 biosatellite and launched on 19 April 2013 on a Soyuz 2-1a rocket from Baikonur Cosmodrome and returned on 19 May 2013 in the Orenburg region of Russia. The BOS habitats were then used to house the GC mice. 13–15 h after return to Earth, study subjects were transported to Moscow, Russia and euthanized. Investigators from University of California, San Diego (UCSD) dissected the calvaria and prepared samples for transport either in RNA later (for quantitative real time PCR analysis) or frozen at −80°C (for lectin staining). The exact details of the BION-M1 experiment is thoroughly reviewed elsewhere (Andreev-Andrievskiy et al., 2014).

### Fenestrae Measurements From Micro-computed Tomography of the Mice Calvaria

The diameter of the skull fenestra were measured from microCT images obtained previously by (Macaulay et al., 2017). The detailed protocol for the preparation of the BION-M1 mice calvaria, volume of interest and the plane used for microCT imaging is provided elsewhere (Macaulay et al., 2017). Briefly, calvariae were moistened with phosphate buffered saline (PBS) and wrapped in tissue paper and scanned with a 9 μm voxel size, an electrical potential of 50 kVp and current of 200 μA, and a 0.5 mm aluminum filter. The parietal section of the calvarial structure flanked rostrally by two frontal bones and caudally by a single bone were imaged on micro-computed tomography scanner, Skyscan 1076 (Bruker μCT, Kontich, Belgium) according to the established guidelines (Bouxsein et al., 2010). A volume of interest (VOI) in the centre of the parietal sections measuring 18 mm^3^, 5.5 mm × 1.2 mm area in the coronal plane, 2.7 mm depth was imaged. The centre was identified by the anterior lamboid and coronal sutures intersection. Histomorphetric measurements of bone structure and thickness was determined SkyScan CT-analyser and DataViewer software. The diameter of the fenestrae on either sides of central sutures were examined qualitatively by drawing a white dotted line around each fenestrae and determining the diameter across the fenestrae.

### Hematoxylin and Eosin Staining

The BION-M1 calvarial samples were extracted from the sacrificed mice after touchdown and transported to the laboratory in 10% formalin. The tissue was then decalcified with 10% ethylenediaminetetraacetic acid solution for 4 weeks. The tissues were dehydrated with graded series of ethanol and embedded in paraffin. Coronal sections were made along the suture region with a thickness of 7 μM and stained with hematoxylin and eosin.

### Lectin Staining of the Calvaria

The calvarial sections from the BION-M1 mice were fixed in 4% PFA in PBS and washed three times with PBS. The sections were then permeabilized in PBT (PBS, 1% BSA and 0.5% Triton X-100) and incubated with FITC-conjugated BS1-B4 (Sigma Aldrich, St. Louis, MO) at 4 C overnight. The tissue sections were then mounted with ProLong Gold Antifade reagent (Invitrogen, Eugene, OR). The parietal cross sections were 7 μm thick and sectioned as duplicates on a single slide. The negative controls were sections without FITC-conjugated BSI-B4. The parasagittal vessels were counted manually and diameter measured throughout the calvarial section (30–38 fields/slide, n = 9 animals for STS-131 and n = 10 animals for BION-M1) by a random-blinded observer in a random manner using the free hand tool of ImageJ software. The pictures were taken with a 40x magnification using a Olympus B×51 FL microscope.

### Quantitative Real Time Polymerase Chain Reaction (qPCR)

The frontal and parietal portion of 5 GC mice and five S F mice from the BION-M1 flight were dissected and stored in RNA post-flight. The broad parietal section (both the bones) of the calvarial tissue samples were then powdered in a homogenizer and transferred to Eppendorf tubes filled with Trizol^®^, after which samples were frozen in liquid nitrogen and prevented from thawing. Following arrival at the distributed laboratories, samples were thawed and treated in Trizol^®^ reagent (Invitrogen; Thermo Fischer Scientific, Inc.) for 30 min to completely disassociate the nucleoprotein complex. After 30 min, the samples were pipetted about 20 times and kept at room temperature (RT) for 2 min 200μL of chloroform was added to Trizol^®^ and agitated for 15 s by hand. The samples were kept at RT for another 2 min. The samples were then centrifuged at 12,000 rpm for 15 min to facilitate phase separation. The aqueous phase was then transferred to an Eppendorf tube containing an equal volume of 70% ethanol. After the total RNA was precipitated, the solution was transferred to an RNA-binding column and RNA purification was carried out according to the manufacturer’s instructions (Purelink RNA, Life technologies). The pure RNA was eluted from the columns in 30 μL of RNase-free water, and the RNA concentration and purity was estimated using nanodrop. Normalized RNA (1 mg/ml) was converted into cDNA using a Qiagen kit with a gDNA wipeout buffer. Reverse transcription was carried out at 42°C for 30 min per manufacturer’s instructions. Resulting cDNA was stored at −20°C or used immediately. The gene expression analysis was carried out using SYBR green (2x QuantiTect SYBR Green PCR, Qiagen) in a 96-well format on an Eppendorf Mastercycler. Primer sequences used for real time PCR are listed in Table 1 and sourced from IDT Technologies. The annealing temperature was standardized at 57°C for 15 s except *RUNX2* and *OCN* for which the annealing temperature was 58°C for 15 s. The results were normalized with *18s RNA* and *GAPDH* housekeeping genes; the experiment was run in triplicates and Livak’s method was used to calculate the difference in expression between SF and GC mice.

**TABLE 1:** Primers for mRNA quantification using qPCR.

Gene Name	5′ to 3′ primer Sequence
*GAPDH*	Sense- GGA​AGG​TGA​AGG​TCG​GAG​T
*GAPDH*	Antisense- CCT​GGA​AGA​TGG​TGA​TGG​G
18sRNA	Sense-CGGCTACCACATCCAAGGAA
*18sRNA*	Antisense-GCGGAATTACCGCGGCT
*VEGFA*	Sense- TTA​CTG​CTG​TAC​CTC​CAC​C
*VEGFA*	Antisense- ACA​GGA​CGG​CTT​GAA​GAT​G
*iNOS*	Sense- GGAGCATCACCCCTGTGT
*iNOS*	Antisense- GGTCTTCCAGGGCTCGAT
*BMP2*	Sense- AAG​GCA​CCC​TTT​GTA​TGT​GG
*BMP2*	Antisense- CAT​GCC​TTA​GGG​ATT​TTG​GA
*RUNX2*	Sense- CCA​CCT​CTG​ACT​TCT​GCC​TC
*RUNX2*	Antisense- ATG​AAA​TGC​TTG​GGA​ACT​GC
*DMP1*	Sense- AGT​GAG​GAG​GAC​AGC​CTG​AA
*DMP1*	Antisense- TCC​CTG​TGG​AGT​TGC​TCT​CT
*OCN*	Sense- GGC​TTA​AAG​ACC​GCC​TAC​AG
*OCN*	Antisense- GAG​AGG​ACA​GGG​AGG​ATC​AA

### Statistics

Gene expression data from quantitative PCR experiments are expressed as a fold difference of the Ct values from housekeeping genes *18s RNA* and *GAPDH*. Differences in gene expression, the number of capillaries, and vessel diameter between SF and GC groups were compared in GraphPad Prism five software using Student’s unpaired t-test. In the absence of a normal distribution, we used the non-parametric Mann-Whitney *u*-test to determine the significance, for which*p < 0.05 was considered significant. All values are mean ± SD and n is the number of animals per group.

## Results

### Changes in Vascular Morphology in Mouse Calvaria after 15 and 30 days of Spaceflight

**FIGURE 2:**
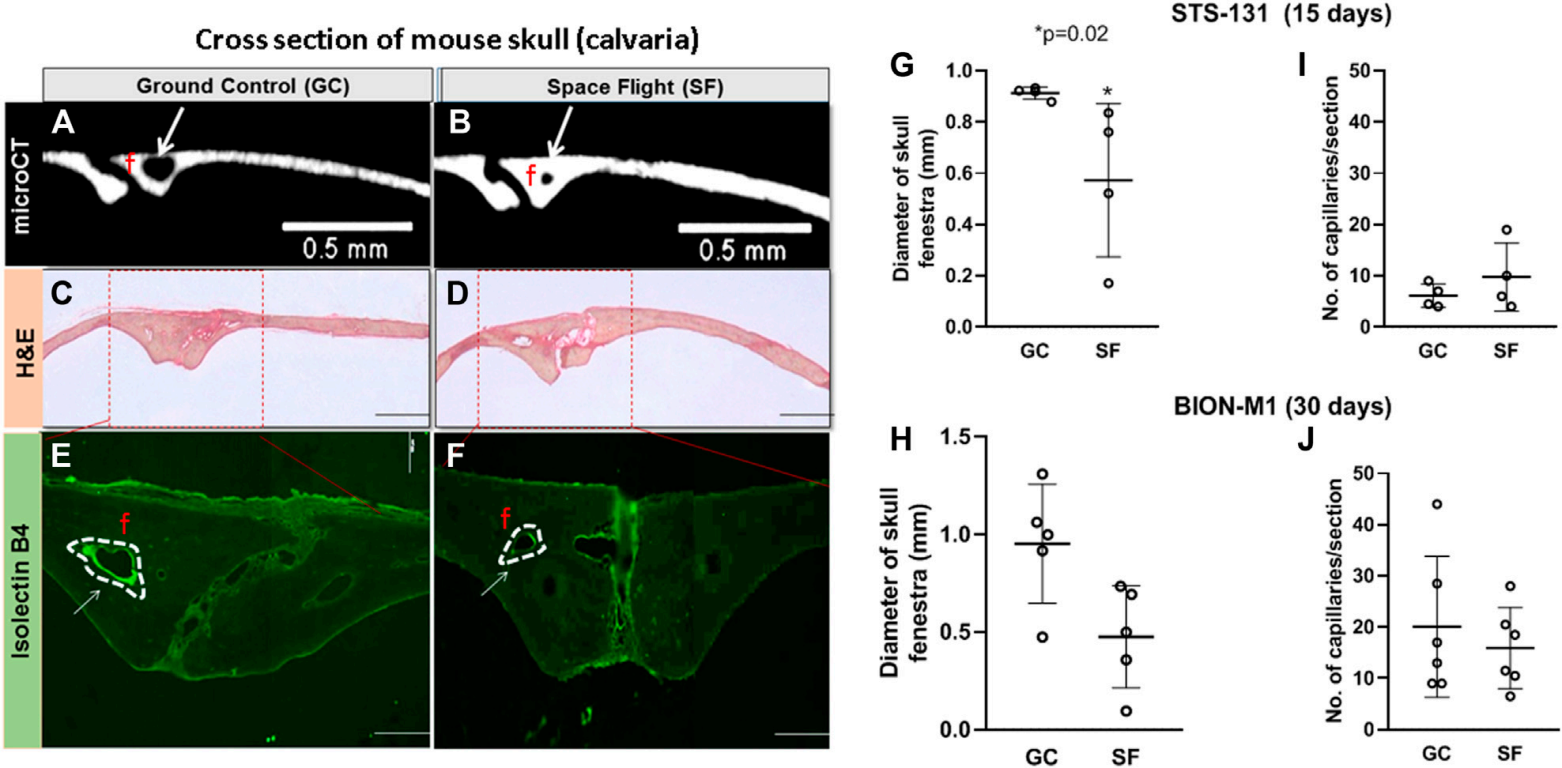
Vascular number and parasagittal capillary size in GC and SF mice calvaria **(A,B)** Coronal sections of μCT images of two parietal plate structures and bone marrow cavities in the midline of the calvaria. The blood vessels (BV) and the edge of the bone plates in the GC and SF mice are shown. The arrows are pointing to the size of the fenestrae **(C,D)** Eosin hematoxylin staining of the frontal section of the parietal region showing two large venules, arteriole, numerous foci, and cell nucleus within the suture surrounded by loose connective tissue **(E,F)** Isolectin-IB4 staining of the parietal sections of the mice calvaria; labeling of both large and small vessels is shown with a dotted white line **(G–J)** Measurements of isolectin-IB4-stained blood vessel diameter and capillary number performed on ≥15–20 microscope images per slide section (n = 2 sections/slide) for a total of eight STS-131 mice exposed to 15 days microgravity (GC, n = 4; SF, n = 4) and 10 BION-M1 mice exposed to 30 days of microgravity (GC, n = 5; SF, n = 5). The values are expressed in mm. The data are represented as mean ± SE, *p = 0.02. The images were obtained with a 4x objective lens and depict fenestrae containing calvarial vessels. Scale bar is 2 mm and the width of the section is 2 mm.

Our previous µCT analysis on STS-131 S F calvaria show that 15 days of spaceflight results in a 30% increase in bone volume in spaceflight mice calvaria compared to control mice (Zhang et al., 2013). However, 30 days of spaceflight in BION-M1 did not show remarkable differences in bone volume and thickness (Macaulay et al., 2017). To determine the spaceflight associated vascular effects in mice calvaria, we first qualitatively compared the size of the parietal fenestrae of BION-M1 previously occupied by the vessels from the BION-M1 µCT micrographs (Macaulay et al., 2017). The bone thickness and bone volume of BION-M1 mice calvarial sections are published elsewhere (Macaulay et al., 2017). The scanned µCT micrographs of calvarial sections from GC and SF mice showed decreases in the size of fenestrae around the parietal sutures apart from the appearance of increases in cross-sectional thickness in the representative SF group compared to GC group (Figure 2A, Panels A and B).

Further qualitative histological examination of the bone trabeculae along the suture showed a number of foci, nucleus and empty spaces left by dead cells in the suture region and the bone plates (Figure 2A, Panel C and D). Next, we quantified parasagittal vessel structural and molecular vascular adaptations to 15-day and 30-day spaceflight by immunostaining capillaries in the sagittal suture section of the mice calvaria with, *Griffonia simplicifolia* lectin isolectin-B4 as a fluorescent endothelial cell marker (Figure 2A, Panels E and F). Manual, double-blinded quantification of Lectin B4-stained parasagittal vessels of the mice calvaria showed a significant increase in vessel numbers in SF mice compared to GC in the STS- 131 mice cohort (GC: 6 ± 2 vessels/mm^2^, SF: 10 ± 6 vessels/mm^2^; n = 4, *p = 0.054, *t*-test and Mann-Whitney u-test) (Figure 2B). The vessel diameters in bone trabeculae are reduced 4-fold in SF compared to GC in STS-131 tissues (15–20 images, two sections/slide, *p = 0.02) (Figure 2A, Panel C). In BION-M1 mice calvaria, there were no significant differences in the diameter or the number of vessels between GC and SF mice (GC: 20 ± 14 vessels/mm^2^, SF: 16 ± 8 vessels/mm^2^; n = 5, *p > 0.05, *t*-test and Mann-Whitney u-test) (Figure 2C). Capillarization is increased and diameter of the vessel is decreased in the STS-131 S F population while no significant change in the vessel numbers or diameter occurs in BION-M1 SF mice.

### Microgravity-Associated Alterations in Calvaria Gene Expression Related to Angiogenesis and Osteogenesis

**FIGURE 3:**
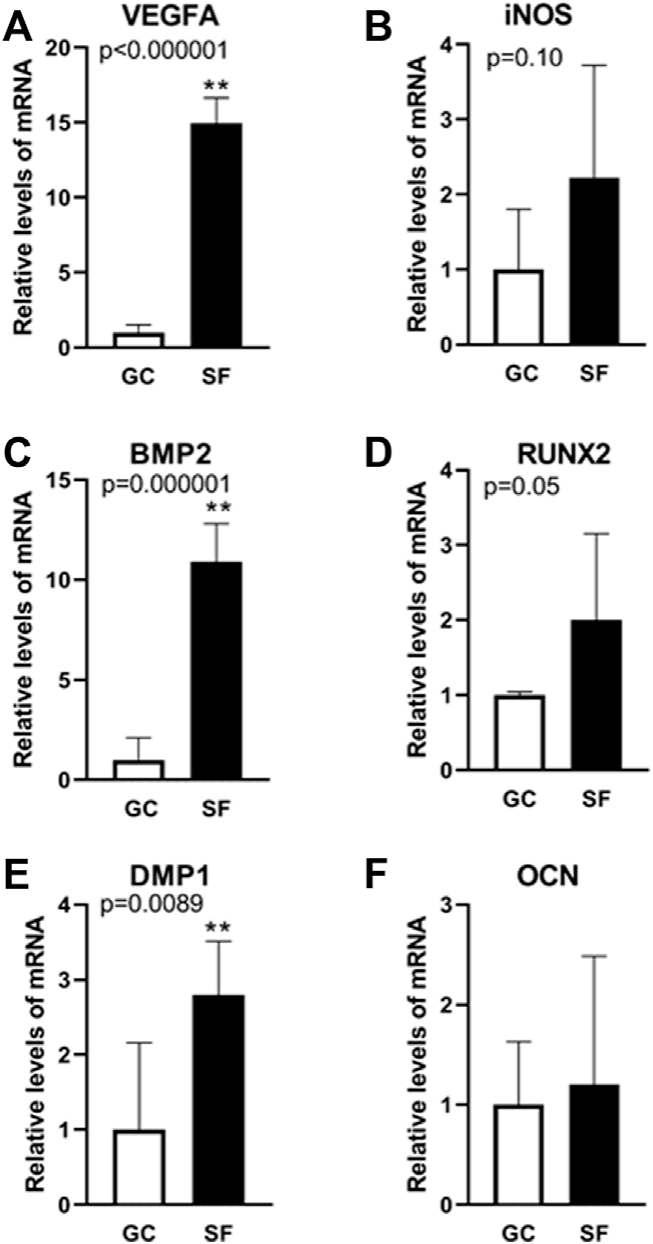
Gene expression analysis related to angiogenesis and osteogenesis in mouse calvaria. Relative mRNA expression levels determined using qPCR analysis of angiogenesis-associated marker genes (VEGFA **(A)**, iNOS **(B)**) and osteogenesis-associated marker genes (BMP2 **(C)**, RUNX2 **(D)**, DMP1 **(E)**, OCN **(F)**) in GC and SF mice calvaria. Values are expressed as mean ± SE. Significant differences are indicated by **p < 0.0001 from an unpaired Student’s t-test followed by Mann-Whitney u-test (n = 6 GC and 6 SF animals).

To determine the effects of cephalad fluid shifts on calvarial angiogenesis and osteogenesis associated gene expression, *VEGFA*, inducible nitric oxide synthase (*iNOS*), *BMP-2*, dentin matrix protein 1 (*DMP1*), *RUNX2* and *OCN* were examined using qPCR in central parietal section of calvaria samples from GC and BION-M1 SF mice. Pro-angiogenic *VEGFA* expression increases 15 folds in SF compared to GC mice (Figure 3, Panel A). Concomitantly, *BMP-2* gene expression increases 11 fold in SF compared to GC (Figure 3, Panel C). *RUNX2*, required for osteoblast differentiation, also increases 2-fold in SF mice compared to GC (Figure 3, Panel D). *DMP1* is involved in the regulation of bone formation and is upregulated in SF mice (Figure 3, Panel F). *OCN* remained unchanged in GC and SF mice. The material was insufficient to analyze *SOST* expression, involved in anabolic activity of bone. In sum, both angiogenic and osteogenic genes in the calvaria are upregulated in response to spaceflight-related fluid shifts.

## Discussion

Representing non-load bearing regions with mouse calvaria and leveraging the STS-131 (15-day) and BION-M1 (30-day) flights, we examined spatial and temporal calvarial vascular remodeling and gene expression related to microgravity exposure. Unloaded regions of the body such as calvaria show an 30% increase in bone volume when exposed to spaceflight conditions compared to ground controls (Zhang et al., 2013), suggesting differential regulation of load bearing and unloaded regions of the body. Previous work has shown impaired cerebral perfusion and blood distribution based on structural modifications of vessels in the brain due to head-ward fluid shifts in mice exposed to microgravity (Sofronova et al., 1985; Taylor et al., 2013). The mechanosensitivity of bone cells to compressive forces and subsequent increase in bone formation in response to mechanical stimuli is known, calvarial adaptations to cephalad fluid shifts, increased intracranial pressure, and cerebrovascular regulation due to microgravity exposure are poorly understood. To address this knowledge gap, we describe for the first time calvarial vascular morphological alterations in mice exposed to STS-131 conditions, along with angiogenesis and osteogenesis-associated calvarial gene expression changes in mice from BION-M1.

Consensus is lacking on the exact effects of microgravity on cerebrovascular regulation as the results from different models are inconsistent. Most of the interpretation of the data is based on patterns derived from other pathologies (Du et al., 2021). Consensus points to fluid redistribution and blood pooling in the brain in the absence of hydrostatic gradients as precipitating the neurological and pathological conditions associated with microgravity. Spaceflight reduces bone mineral density in the lower load-bearing musculoskeletal regions compared to non-load bearing regions such as the skull (LeBlanc et al., 2000; Vico et al., 2000; Zhang et al., 2013). Mouse skull is a rarely studied structure in spite of its abundance of blood and lymphatic vessels, as well as its structural and functional roles during homeostasis and repair. Calvarial thickness and suture growth is age-dependent and progressive (Alberius and Johnell, 1990). The suture section of the calvarial parietal bone consists of the maximum density of the capillaries which support bone development and is therefore suitable for studying vessel morphology and number. The suture in the calvaria is heavily vascularized with small central vessels (Alberius and Johnell, 1990). Lectin binding to the glycoconjugates of endothelial cells plasmalemma allow for a simple method for the visualization of morphology. Therefore, we used lectin stain to label calvarial blood vessels.

Blood vessel diameter is up- or downregulated depending on the intensity and direction of blood flow. Correspondingly, our study demonstrated reductions in calvarial blood vessel diameter in STS-131 S F mice as a response to increase in demands for blood flow and bone volume. Our data showed differential profiles for vessel diameters and numbers in the calvarial sections of mice included on STS-131 (15-day) or BION-M1 (30-day). In STS-131 mice, the vessel diameter was reduced and capillarization increased, a trend suggesting calvarial vessel remodeling and capillarization in the bone trabeculae concomitant to increase in calvarial bone mass identified in early study (Zhang et al., 2013).

Clinical and experimental studies have demonstrated a causal link between expression of angiogenic markers and bone formation (Cackowski et al., 2010; Moser et al., 2018). During injury or stress, resident suture stem cells secrete VEGF and recruit blood vessels to facilitate repair; VEGF stimulates the proliferation of osteoblast and increased VEGF levels are observed at the proliferative stage of bone repair, and this vascularization is essential for tissue recovery and repair. Insufficient or incongruous formation of bone blood vessels leads to irregular bone formation and increased risk of bone fractures (Stegen et al., 2015). The regeneration of vascular supply through vascularization in parallel with osteogenesis is central in the formation of the new functional bone (Amini et al., 2012). Alterations in VEGFA expression correlates with the number of vessels in spaceflight calvaria in the STS-131 flight mice (Figure 2B).

Local application of exogenous recombinant BMP2 completely regenerates calvarial defects in a rodent model and is important in tissue healing and restoration. In humans, BMP2 recombinant protein can regenerate mandibular continuity and cleft palate defects (Boyne, 2001; Dickinson et al., 2008; Herford and Boyne, 2008). BMP-2 addition dramatically increases Osteocalcin (OCN) which in turn is responsible for the pro-osteogenesis function of BMP2. BMP2 induces osteoblast differentiation through *RUNX2* expression which directly regulates osteocalcin expression to increase bone formation. *RUNX2* is a global regulator of osteogenesis and is crucial for regulating the expression of bone-specific genes. Autocrine BMP production is necessary for the Runx2 transcription factor to be active. Runx2 executes and completes BMP2 signaling for osteoblast differentiation. The co-ordinated activity of Runx2 and BMP2 activated SMADS are critical in bone remodeling and skeleton formation. Taken together, endogenous BMP signaling enhances bone formation and regulation of BMP activity in the postnatal stages is critical for normal bone formation. While acknowledging that there may be site specific and bone dependent differences described by others (Quarto et al., 2010; Kwan et al., 2011; Niziolek et al., 2011), we show that the parietal region of the mice calvaria are sensitive to fluctuations in fluid shifts and results bone vascular responses.

Microgravity-mediated cephalad fluid shift results in high intracranial pressure which may affect vessel morphology. Cerebral vasomotor and mechanical properties, deficiencies in femoral muscle regeneration and defective immune response occurs after 30 days of BION-M1 spaceflight (Sofronova et al., 1985; Andreev-Andrievskiy et al., 2014; Markina et al., 2018; Radugina et al., 2018; Tascher et al., 2019). Contrary to this finding, our µCT data of the BION-M1 mice suggests that the calvarial bone structure remain unchanged. However the gene expression of sclerostin, a potent inhibitor of bone formation, is higher in the spaceflight group based on our previous results (Macaulay et al., 2017) implying the possibility of both anabolic and catabolic activity of sclerostin. This is in contrast to previous reports that show that loss of sclerostin increases cranial bone growth and regeneration and sclerostin antibodies are used to increase bone mass in clinical trials of osteoporosis and osteogenesis imperfecta (Kang et al., 2018; Scheiber et al., 2019) suggesting that other mechanisms may be involved in bone formation.

One of the mechanisms of bone regeneration is the recruitment of stromal and hematopoietic precursors from the bone marrow (Markina et al., 2018). The proliferative capacity of the bone marrow stromal and hematopoietic precursors decreases in the BION-M1 mouse population cohort of mice (Markina et al., 2018) which may explain our findings that the vessel diameter and number of vessels are unchanged in BION-M1 compared to STS-131 (Figure 2). The molecular mechanisms underlying these physiological adaptations of calvarial vessels and bone to cephalad fluid shifts remain poorly understood.

## Conclusion

This work describes microgravity-associated alterations in pro-angiogenic and pro-osteogenic gene expression in an unloaded bone region in two wild-type study populations. These results suggest skull vasculature and bone as a sensor for gravity and cephalad fluid shifts, outcomes that highlight the importance of the development of countermeasures for long-duration spaceflight. An in-depth, gender-based study of mouse calvaria that includes genomic, proteomic and metabolomic components will further characterize pathways involved in the increase in calvaria growth seen in study populations exposed to microgravity. Future study will also examine the behavior of BMP-2 gene expression levels post-flight, and whether these levels return to baseline prior to euthanasia. These results also suggest that key molecules associated with angiogenesis (VEGFA) and osteogenesis (BMP-2) genes are modulated in BION-M1 mice calvaria exposed to 30 days of microgravity. We suggest that VEGFA and BMP-2 genes are gravity sensors in mouse calvaria. The finding is relevant in the development of countermeasures for prolonged crewed spaceflight and to understand the molecular and structural adaptations of bone and vasculature to the microgravity environment. Lastly, there is a need to monitor the entire astronaut skeletal system on a meta-scale and in a spatio-temporal manner; this type of inquiry will leverage the site-specific, heterogenic skeletal responses and the sufficiency of short-term microgravity exposure to induce upregulation of angiogenic and osteogenic gene expression.

## Limitations

The authors recognize several limitations for this study as well as associated opportunities for ongoing study. First, comparisons between study subjects required analysis between genders as well as between varying durations of microgravity and carbon di-oxide exposure. While no evidence of significant skull size differences between the male and females exists, contrasting data from STS-131 females and BION-M1 males will need to be controlled in future work. Second, suture lymphatic vessels were recently identified that may have also accepted lectin stain alongside venules and arterioles. Third, BION-M1 post-flight “recovery” was comprised of 13–15 h needed to transport study subjects from the landing site to the lab for euthanasia. This period of reloading was not controlled for and may have contributed to the gene expression changes seen in the flight animals. Fourth, several whole animals were excluded from data collection due to lack of tissue section integrity on slides. Fifth, we were unable to perform further mechanistic studies using knockout mice due to restricted payload space, prohibitive costs, and other technical challenges. Lastly, due to the limitations in tissue size, we were not able to confirm the protein expression of VEGFA and BMP-2 using immunoblotting. immunostaining, and localization of the proteins using imaging techniques. Future work is planned to examine the microgravity-induced modulation of mouse calvaria at epigenetic, gene, and protein levels. Trends in the heterogeneous skeletal and vascular adaptations dependent on the location and load-bearing properties of the tissue, in addition to angiogenic and osteogenic gene expression, are intriguing. Ongoing study is intended to address the benefit or detriment of this effect.

## Data Availability

The raw data supporting the conclusions of this article will be made available by the authors, without undue reservation.
